# Underuse of cardiorenal protective agents in high-risk diabetes patients in primary care: a cross-sectional study

**DOI:** 10.1186/s12875-022-01731-w

**Published:** 2022-05-24

**Authors:** Robin Hao, Tyler Myroniuk, Taylor McGuckin, Donna Manca, Denise Campbell-Scherer, Darren Lau, Roseanne O. Yeung

**Affiliations:** 1 Department of Medicine, University of Alberta, 13-103 Clinical Sciences Building, 11350-83 Avenue, Edmonton, Alberta T6G 2G3 Canada; 2 Department of Public Health, University of Missouri, 510 Lewis Hall, Columbia, MO 65211 USA; 3 Physician Learning Program, Faculty of Medicine and Dentistry, University of Alberta, 2-590 Edmonton Clinic Health Academy, 11405-87 Ave NW, Edmonton, Alberta T6G 1C9 Canada; 4 Department of Family Medicine, University of Alberta, Suite 5-16, University Terrace, 833 112 Street, Edmonton, Alberta T6G 2T4 Canada; 5 Division of Endocrinology & Metabolism, University of Alberta, 2-590 Edmonton Clinic Health Academy (ECHA) 11405-87 Ave NW, Edmonton, Alberta T6G 1C9 Canada

**Keywords:** Diabetes mellitus, Primary care, SGLT2 inhibitors, GLP1-receptor agonists, Antihyperglycemic agents, Electronic medical records

## Abstract

**Background:**

Sodium-glucose cotransporter-2 inhibitors (SGLT2i) and glucagon-like peptide-1 receptor agonists (GLP-1RA) have shown benefits in patients with diabetes and cardiovascular disease (CVD), heart failure (HF), and chronic kidney disease (CKD).

**Objective:**

We assessed benchmark outcomes (Hemoglobin A1c, LDL-C, and blood pressure), identified the prevalence of cardiorenal indications for SGLT2i and GLP-1RA, and compared prescribing rates of GLP1-RA and SGLT2i in those with and without cardiorenal indications.

**Methods:**

We analyzed data from January 2018–June 2019 for 7168 patients with diabetes using electronic medical records from the Northern Alberta Primary Care Research Network, a regional network of the Canadian Primary Sentinel Surveillance Network (CPCSSN). Patients with and without cardiorenal comorbidities were compared using descriptive statistics and two proportion Z tests.

**Results:**

Hemoglobin A1c ≤ 7.0% was met by 56.8%, blood pressure < 130/80 mmHg by 62.1%, LDL-C ≤ 2.0 mmol/L by 45.3% of patients. There were 4377 patients on glucose lowering medications; metformin was most common (77.7%), followed by insulin (24.6%), insulin secretagogues (23.6%), SGLT2i (19.7%), dipeptidyl peptidase-4 inhibitor (19.3%), and GLP-1RA (9.4%). A quarter of patients had cardiorenal indications for SGLT2i or GLP-1RA. Use of SGLT2i in these patients was lower than in patients without cardiorenal comorbidities (14.9% vs 21.2%, *p* < 0.05). GLP-1RA use in these patients was 4.6% compared with 11% in those without cardiorenal comorbidities (*p* < 0.05).

**Discussion:**

Contrary to current evidence and recommendations, SGLT2i and GLP1-RA were less likely to be prescribed to patients with pre-existing CVD, HF, and/or CKD, revealing opportunities to improve prescribing for patients with diabetes at high-risk for worsening cardiorenal complications.

**Supplementary Information:**

The online version contains supplementary material available at 10.1186/s12875-022-01731-w.

## Key messages

1. SGLT2i and GLP-1RA have organ-preserving benefits in patients with type 2 diabetes and chronic kidney disease, cardiovascular disease, and/or heart failure.
2. These medications were prescribed to fewer diabetes patients with cardiorenal disease than those without these comorbidities in a primary care setting.
3. There is an urgent need to optimize prescribing given a quarter of primary care diabetes population has cardiorenal disease with low uptake of these medications.

## Background

Novel therapeutics that reduce mortality and morbidity have transformed diabetes management. However, there are significant barriers to their use in clinical practice to the detriment of patients and the healthcare system [1]. In patients with prior cardiovascular disease, both glucagon-like peptide-1 receptor agonists (GLP-1RA) and sodium–glucose cotransporter 2 inhibitors (SGLT2i) have shown reductions in major adverse cardiac events (nonfatal myocardial infarction, nonfatal stroke and cardiovascular death) [2, 3]. In a recent meta-analysis by Zelniker et al., SGLT2i have demonstrated an overall reduction of major adverse cardiovascular events by 11%, hospitalization for heart failure by 23%, and progression of renal disease by 45% [2]. Similarly, Kristensen et al. found that GLP1-RA have cardiorenal protection with reduction of major adverse cardiac events outcomes by 12%, hospital admission of heart failure by 9%, and broad composite kidney outcomes by 17% [3]. In the 2018 Clinical Practice Guidelines, Diabetes Canada began recommending these agents specifically for high-risk patients with known cardiovascular disease (CVD) and chronic kidney disease (CKD) [4]. Updated 2020 Clinical Practice Guidelines further reinforce this message and expand to include patients with heart failure (HF) and those with 2 or more significant cardiovascular risk factors such as smoking, hypertension, dyslipidemia, or obesity [5].

With diabetes affecting 3.7 million Canadians, where 25% have established kidney disease and 20% have cardiovascular disease, there is a pressing need to reduce the complications of diabetes through optimal use of these new medications [6, 7]. From 2015 to 2017 in Alberta, Tonelli et al. described population characteristics of patients with diabetes and CKD, noting an overall prescribing rate of 7.6 and 2.4% for SGLT2i and GLP1-RA, respectively [8]. With changes to Clinical Practice Guidelines and emerging evidence for clinical benefit, there remains a gap in uptake of SGLT2i and GLP-1RA in high- risk diabetes populations.

## Methods

### Aim, design, and setting

In this cross-sectional study of patients in Alberta primary care, we examine the characteristics of patients with diabetes, the prevalence of cardiorenal indications for SGLT2i or GLP- 1RA, and the rates of SGLT2i and GLP-1RA prescribing in these high-risk patients relative to other patients with diabetes.

We used data from the Northern Alberta Primary Care Research Network (NAPCReN). NAPCReN is 1 of the 13 primary care research networks in Canada that contribute data to the Canadian Primary Care Sentinel Surveillance Network (CPCSSN), a Canadian electronic medical record (EMR)-based surveillance system [9]. NAPCReN captures extensive demographic and clinical data from EMRs [10].

### Participant characteristics

We included adult patients ≥18 years old with diabetes mellitus who visited an Alberta primary care clinic submitting data to NAPCReN between January 1, 2018 and June 30, 2019. Diabetes mellitus was defined using the established CPCSSN definition, which has been validated with a sensitivity of 95.6% and specificity of 97.1% (Additional file 1) [11, 12]. Patients included either had a history of diabetes, as captured in the CPCSSN, or a hemoglobin A1c (HbA1c) ≥ 6.5% between January 1, 2018 and June 30, 2019. Although CPCSSN’s definition included HbA1c of ≥7%, we modified it to include HbA1c ≥ 6.5% as per current Diabetes Canada diagnostic criteria [13]. Thus, the validated definition included any type of diabetes mellitus in our sample. Utilization of CPCSSN database and International Classification of Diseases, Ninth Revision (ICD-9) codes to differentiate between type 1 and type 2 diabetes mellitus have not been validated, and therefore, this was not pursued. Ethics approval was obtained from the Health Research Ethics Board – Health Panel at the University of Alberta (Pro00095694). The need for written informed consent was waived by the Health Research Ethics Board - Health Panel ethics committee due to retrospective nature of the study*.* This project was conducted by the Physician Learning Program with financial support from the Government of Alberta. The views expressed herein do not necessarily represent the official policy of the Government of Alberta.

### Measurements and outcomes

Data were obtained on patient demographic and physical examination variables including blood pressure, laboratory results (estimated glomerular filtration rate [eGFR], HbA1c, albuminuria, albumin- creatinine ratio [ACR], low-density lipoprotein cholesterol [LDL-C]), smoking status, prescription medications, and comorbidities. For patients with more than one clinic visit, the most recent values were used for blood pressure, LDL-C, and creatinine. We categorized patient data following these guideline-concordant targets: blood pressure (< 130/80 mmHg), LDL-C (≤2.0 mmol/L), and HbA1c (≤7%) [14, 15].

High-risk patients were identified by prior diagnoses of atherosclerotic cardiovascular disease, HF, and CKD. Cardiovascular comorbidities were identified using ICD-9 codes, specifically identifying CVD, HF, and procedural codes for operations of the heart vessels or endovascular procedures (Additional file 2).

Patients were classified as having CKD based on eGFR and albuminuria based on Kidney Disease: Improving Global Outcomes (KDIGO) staging guidelines [16]. eGFR was derived from CKD Epidemiology Collaboration equation based on the patient’s creatinine and characteristics. Albuminuria was defined by ACR ≥ 30 mg/mmol. KDIGO staging guidelines were used with suggested stages: eGFR ≥90 mL/min/1.73 m^2^ and albuminuria (stage 1), 60 to 89 mL/min/1.73 m^2^ and albuminuria (stage 2), 45 to 59 mL/min/1.73 m^2^ (stage 3a), 30 to 44 mL/min/1.73 m^2^ (stage 3b), 15–29 mL/min/1.73 m^2^ (stages 4), and < 15 mL/min/1.73 m^2^ (stage 5).

All diabetes medications were retrieved from NAPCReN based on Anatomical Therapeutic Chemical codes and categorized by therapeutic classes (dipeptidyl peptidase-4 inhibitor [DPP4i], GLP-1RA, insulin, insulin secretagogues, metformin/biguanides, SGLT2i, and thiazolidinediones). These data captured pre-existing medications, as well as prescription refills and new prescriptions of antihyperglycemic agents. Patients were considered to have been prescribed SGLT2i or GLP-1RA if they had one or more prescriptions between January 1, 2018 to June 30, 2019.

### Statistical analysis

Demographic data are presented as mean (standard deviation [SD]) or n (%). Prescribing rates of medication classes were reported as simple proportions stratified by HbA1c and by cardiorenal comorbidity groups. We performed between-group analyses comparing prescribing rates of SGLT2i, GLP-1RA, and other medications among those with cardiorenal co-morbidities versus those without these co-morbidities using two-proportion Z-tests. Given that current SGLT2i monographs do not recommend for initiation in patients with eGFR < 30 mL/min/1.73 m^2^, we completed a sensitivity analysis excluding such patients from SGLT2i prescribing rates. All programming and analyses were conducted using Stata 16.

## Results

### Patient characteristics

Between January 2018 and June 30, 2019, we identified 7168 patients with diabetes, 51.1% of whom were male, with mean age of 65 ± 15 years (Table 1). The mean HbA1c was 7.2 ± 1.5%. HbA1c was not recorded in 15.6% of the population. 60.6% of those with documented body mass index (BMI) were considered to be living with obesity (BMI > 30 kg/m^2^). Of those with a documented HbA1c, 9.9% had a most recent HbA1c > 9%, while another 10.4% had HbA1c between 8 and 9%. Regarding blood pressure, 62.1% of the patients met guideline targets of < 130/80 mmHg, while 42.2% of patients did not have recently documented blood pressure. The target of LDL-C of 2.0 mmol/L was achieved by 45.3% of the patients with documented lipid results.

**Table 1:** Patients prescribed each medication for those with CVD/HF and/or CKD and those without

	CVD/HF and/or CKD	No CVD/HF/CKD	Total N with medication prescribed
**Medication**	*N (% of comorbidity group)*		
SGLT2i*	161 (14.9)	701 (21.2)	862
GLP-1RA*	50 (4.6)	363 (11.0)	413
Metformin/Biguanide	828 (76.8)	2573 (78.0)	3401
Insulin secretagogues*	304 (28.2)	729 (22.1)	1033
DPP4i*	240 (22.3)	603 (18.3)	843
Insulin*	330 (30.6)	747 (22.6)	1077
**Total in comorbidity group**	**1078**	**3299**	**4377**

Used to indicate a significant difference between both comorbidity groups at *p* < 0.05

Overall, the prevalence of patients with CVD or HF in our sample was 9.5% (*n* = 680). The prevalence of CKD patients was 17.9% (*n* = 1281). KDIGO staging of CKD patients demonstrated a large predominance of CKD stage 3 a/b that included 83.8% of CKD sample. Patients with any CVD, HF, or CKD comprised 24.1% (*n* = 1727) of the sample.

#### Medication prescribing

**Fig. 1:**
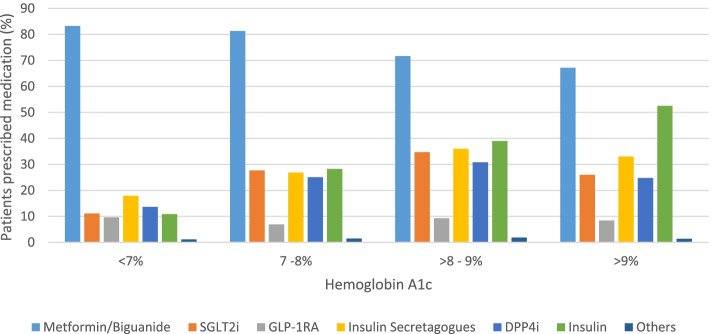
Glucose lowering medications used in patients with diabetes and grouped by HbA1c. Patients using metformin/DPP4i combinations were counted in both groups. The “Other” group included alpha-glucosidase inhibitors and thiazolidinediones

There were 4377 patients (61.1% of the diabetes population) with at least 1 glucose lowering medication prescribed between January 1, 2018 and June 30, 2019; metformin was the most commonly used agent (77.7%), followed by insulin (24.6%), insulin secretagogues (23.6%), SGLT2i (19.7%), DPP4i (19.3%), GLP-1RA (9.4%), and thiazolidinediones (1.1%). When stratified by HbA1c above and below 9%, SGLTi and GLP-1RA were both prescribed in lower proportions when HbA1c was over 9% (Fig. 1).

**Fig. 2:**
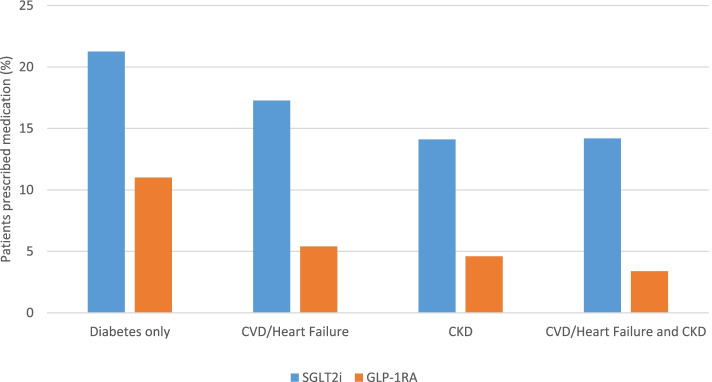
SGLT2i and GLP-1RA use in patients with diabetes and without other cardiorenal comorbidities (CVD, HF, and/or CKD). CVD/HF group included patients with defined CVD (ischemic heart disease, cerebrovascular disease, and/or peripheral vascular disease) and/or HF, which were obtained from ICD-9 codes (See Additional file 2 for details)

For patients with CKD, contrary to recommendations, those with CKD had lower overall SGLT2i (14.1%) and GLP-1RA (4.6%) usage (Fig. 2). Notably, CKD patients had higher proportions on insulin (33.0%), insulin secretagogues (27.6%), and DPP4i (21.3%) compared to patients with diabetes without CKD. Similarly, patients with CVD/HF had lower usage of SGLT2i (17.3 vs. 21.3%) and GLP-1RA (4.6 vs. 11.0%). These patients also had increased utilization of insulin (27.0%), insulin secretagogues (29.9%), and DPP4i (22.3%) compared to those with diabetes without CVD/HF. When all patients with cardiorenal comorbidities (i.e. CVD, HF, and/or CKD) were compared to those patients without cardiorenal comorbidities, (Table 2), they exhibited lower use of SGLT2i (14.9% vs 21.2%, *p* < 0.00001) and lower use of GLP-1RA (4.6% vs 11.0%, *p* < 0.00001), compared to those without comorbidities (Table 2, Fig. 2).

**Table 2:** Baseline characteristics of study population

Patient Characteristics	***N*** (%)
**GENERAL DEMOGRAPHICS**	
Patients identified with diabetes	7168
Mean age (SD)	64.8 (14.7)
Male sex	3663 (51.1)
BMI (kg/m^2^) (*n* = 4256)	
< 25	501 (11.8)
25–30	1176 (27.6)
> 30	2579 (60.6)
**LABS**	
Hemoglobin A1c (*n* = 6047)	
≤ 7%	3436 (56.8)
> 7 - ≤ 8%	1383 (22.9)
≥ 8 - ≤ 9%	631 (10.4)
> 9%	597 (9.9)
Not Available	1121 (15.6)
Blood Pressure (*n* = 4141)	
< 130/80 mmHg^a^	2571 (62.1)
≥130/80 mmHg	1570 (37.9)
Not available	3027 (42.2)
LDL-C (*n* = 3767)	
≤ 2 mmol/L	1708 (45.3)
> 2 mmol/L	2059 (54.7)
Not Available	3, (47.4)
**COMORBIDITIES**	
CVD or Heart Failure	680 (9.5)
CKD	1281 (17.9)
Stage 1^b^	19 (1.5)^c^
Stage 2	54 (4.2)
Stage 3a/b	1073 (83.8)
Stage 4	111 (8.7)
Stage 5	24 (1.9)
CVD/Heart failure *and* CKD^d^	234 (3.3)
**MEDICATION USE**	
Anti-hyperglycemic use	
Yes	4377 (61.0)
No/Not Available	2791 (39.0)
Insulin use (*N* = 7168)	
Yes	1077 (15.0)
No/Not Available	6091 (85.0)

*BMI* Body mass index, *LDL-C* Low-density lipoprotein cholesterol. Insulin use includes fast-acting insulin analogues, fast-acting insulin, intermediate-acting insulin, long-acting insulin analogues, mixed human insulin, mixed insulin analogues, combinational insulin based Anatomical Therapeutic Chemical (ATC) codes

^a^Thresholds based on Hypertension Canada and Diabetes Canada Clinical Practice Guidelines

^b^CKD stages detailed in methods section

^c^Proportion of the 1281 patients with CKD

^d^Patients already enumerated above – thus the total number of patients with CVD/HF or CKD is *n* = 1727

### Sensitivity analyses

Our sample included 135 patients with eGFR < 30 mL/min/1.73 m^2^. Exclusion of these patients did not impact the results appreciably.

## Discussion

Contrary to evidence-based indications, our findings reveal the relative underuse of cardiorenal protective agents in high-risk diabetes patients in the primary care setting in Alberta, where less than 20% of patients with established cardiorenal disease are prescribed organ-preserving medications. This is of urgent concern given that high-risk patients with established cardiorenal disease account for a quarter of the diabetes population in primary care.

This treatment gap exists despite a higher degree of SGLT2i (19.7%) and GLP-1RA (7.6%) utilization than previously described in Alberta [8], and is not unique to Alberta. Vencio et al. reported similar rates of under-prescribing of these protective medications across 13 countries in 2019, with only 21.9% of the eligible high-risk population receiving SGLT2i or GLP1-RA [17]. In a U.S. nationwide cohort study, McCoy et al. noted a similar trend, where SGLT2i were more often prescribed in lower risk patients with fewer comorbidities [18]. These findings fly in the face of robust and mounting evidence demonstrating the efficacy of SGLTi and GLP1-RA in reducing progression of diabetes complications, and the recommendations of Diabetes Canada [4] and other major professional organizations [14, 19, 20]. Moreover, our findings show medications without equivalent evidence for cardiorenal protection (i.e. sulfonylureas, DPP4i, and insulin) continue to be more frequently prescribed in these high-risk patients.

Known barriers to optimal prescribing include access to newer medications, clinician knowledge gaps, patient preferences, adherence, and medical contraindications [21]. Despite publicly funded healthcare, medication coverage remains a major barrier in Canada [22]. Some major insurers require completion of special authorization forms to encourage appropriate prescribing. For instance, in order to qualify for SGLTi and GLP1-RA coverage under the Alberta Blue Cross benefits, prescribers must have trialed metformin for a minimum of 6 months, trialed sulfonylureas, and insulin before qualifying for coverage. These special authorization processes often lag behind the evidence and add additional administrative burden on prescribers, further delaying initiation and intensification of treatment in high-risk populations [23].

When we examined clinical benchmarks of diabetes control, HbA1c, blood pressure, and LDL-C targets were met by 56.8%, 62.1%, and 45.3% of those in whom measurements were available, similar to studies conducted in Ontario between 2010 and 2013 despite changes in diabetes management and introduction of new medications [24, 25]. Notably, we observed that almost half of patients with diabetes in our study were missing recent measurements for blood pressure and lipid measurements. This may be limited by only using LDL-C for assessment, as both ApoB and non-high density lipoprotein cholesterol are reasonable alternatives, preventing us from accurately concluding on the trends of lipid management. Another reason for the lack of lipid measurements may be reflected by different guideline recommendations; Canadian family practice guidelines suggests not repeating lipid measures when on statin therapy and not targeting specific LDL-C levels, which contrast with other professional guideline recommendations to titrate therapy to target [26]. Guideline variations make quality improvement in diabetes care more complicated. Since research shows that patients with diabetes and good risk factor control (HbA1c, LDL, blood pressure, albuminuria, nonsmoking status have similar mortality and cardiovascular events as people without diabetes [27], it is all the more important to dig deeper into these issues to improve diabetes care.

Suboptimal prescribing in high-risk populations is also seen in angiotensin-converting enzyme inhibitors, angiotensin II receptor blockers, beta-blockers, and statins [28–30]. Despite decades of evidence supporting benefit for these medications, prescribing for cardioprotection in patients with diabetes remains low (e.g.: 8–57% of CVD patients in the US) [31], even though numerous studies have found that those with the lowest rates of prescribed beta-blockers and ACEi/ARBs are at the highest risk of death [28–30]. Multiple explanations for this paradoxical risk/treatment mismatch have been proposed, including decreased attention for individual disease management in complex patients with multimorbidity [32], clinician uncertainty of risk vs benefit in high risk populations, discomfort discussing adverse events of newer and less familiar medications, and belief that the benefits observed in clinical trials do not translate into clinical practice [33].

Limitations of our study relate to the use of EMR-based data. Medication data represents prescriptions entered by primary care providers, where we could not account for individuals who did not fill their prescriptions. The medication data did not include prescriptions by hospitals or other specialists; however, these prescriptions are usually entered into the EMR when patients are next reviewed in primary care. Primary care provides most diabetes care in Canada [34], and previous analyses of medications from similar primary care-based networks in Ontario has shown good overall correspondence with expected administrative data-based prescribing patterns [35]. Recognizing that there are occasionally reasons *not* to prescribe SGLT2i in certain patients, we excluded patients in stage 4 or 5 CKD from comparative analysis. Other contraindications, apart from kidney impairment, are rare [36]. Another limitation was our inability to reliably distinguish between type 1 and type 2 diabetes, but given that type 2 diabetes represents more than 90% of diabetes diagnoses, the findings remain informative [37]. ICD-9 codes for classification of comorbidities such as CVD and HF generally have high positive predictive value of 96–97% but lower sensitivity, and it may be that patients with more severe cardiovascular comorbidities were more likely to be identified with our methods [38]. That being said, NAPCReN data provides a perspective relevant to primary care by using information family physicians would have available to them at the point of care, and patients identified as having cardiorenal comorbidities have all the more reason to be prescribed therapy with end-organ benefits.

## Conclusion

Prescribing rates of SGLT2i and GLP1-RA in diabetes care have increased modestly compared to previous studies in Canada, but remain paradoxically lower in patients with diabetes and established cardiorenal comorbidities, despite robust evidence of safety and effectiveness in these populations. The reasons for reluctance to prescribe SGLT2i and GLP-1RA in patients with cardiorenal comorbidities remain complex and may involve factors such as medication coverage, uncertainty of potential harms, and discomfort in medically complex patients. Further efforts are needed to understand the reasons for the observed trends, and to partner with primary care clinicians to devise means of maximizing the benefits of newer agents in patients with diabetes at elevated cardiorenal risk.

## Supplementary Information

**Additional file 1.** CPCSSN definition of Diabetes Mellitus.

**Additional file 2.** ICD 9 numbers and names of diagnosis or procedures used to capture cardiovascular disease and heart disease.

## Data Availability

The data that support the findings of this study are available from Northern Alberta Primary Care Research Network (NAPCReN) but restrictions apply to the availability of these data, which were used under license for the current study, and so are not publicly available. Data can be requested through Dr. Donna Manca, Director of NAPCReN.

## Abbreviations

SGLT2i: Sodium-glucose transport protein 2 inhibitors
GLP-1RA: Glucagon-like peptide 1 receptor agonists
CVD: Cardiovascular disease
CKD: Chronic kidney disease
HF: Heart failure
NAPCReN: Northern Alberta Primary Care Research Network
CPCSSN: Canadian Primary Care Sentinel Surveillance Network
EMR: Electronic medical record
HbA1c: Hemoglobin A1c
eGFR: Estimated glomerular filtration rate
ACR: Albumin to creatinine ratio
LDL-C: Low-density lipoprotein cholesterol
ICD-9: International Classification of Diseases, Ninth Revision
KDIGO: Kidney Disease: Improving Global Outcomes
DPP4i: Dipeptidyl peptidase-4 inhibitor
BMI: Body mass index
