# Supplementary material for: Underuse of cardiorenal protective agents in high-risk diabetes patients in primary care: a cross-sectional study
Source: BMC Prim Care. 2022 May 24;23:124. doi: 10.1186/s12875-022-01731-w (PMC9128222; doi:10.1186/s12875-022-01731-w)
Supplement: Supplementary file 1 — Additional file 1. CPCSSN definition of Diabetes Mellitus. [file 12875_2022_1731_MOESM1_ESM.docx]

**Additional File 1: CPCSSN definition of Diabetes Mellitus**


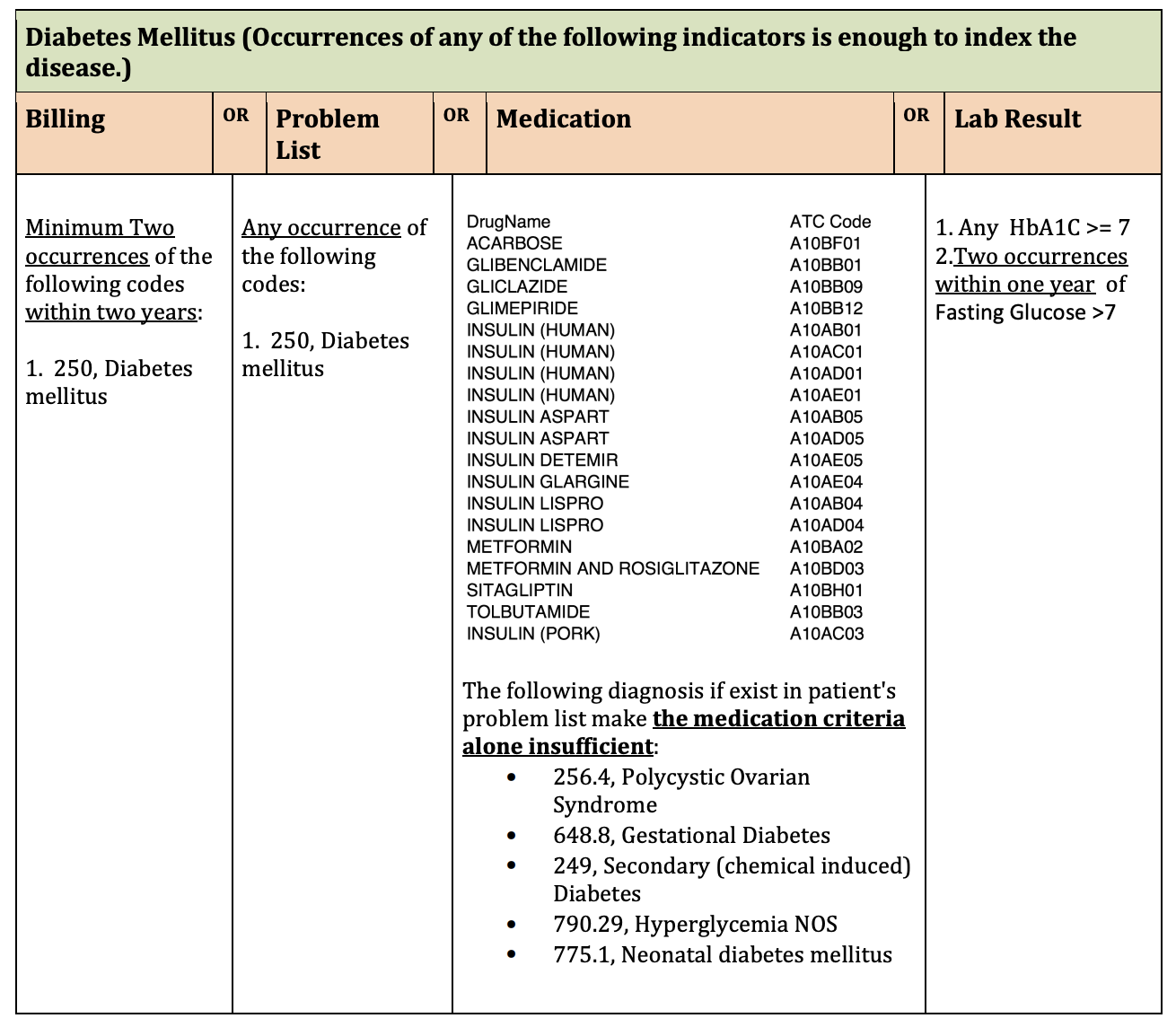


Table adapted from: Williamson, T., et. al,. CPCSSN Disease Definitions: Canadian Primary Care Sentinel Surveillance Network (CPCSSN). June 15, 2014. URL: <http://cpcssn.ca/research-resources/case-definitions>
