# Supplementary material for: Underuse of cardiorenal protective agents in high-risk diabetes patients in primary care: a cross-sectional study
Source: BMC Prim Care. 2022 May 24;23:124. doi: 10.1186/s12875-022-01731-w (PMC9128222; doi:10.1186/s12875-022-01731-w)
Supplement: Supplementary file 2 — Additional file 2. ICD 9 numbers and names of diagnosis or procedures used to capture cardiovascular disease and heart disease. [file 12875_2022_1731_MOESM2_ESM.docx]

**Additional File 2: ICD 9 numbers and names of diagnosis or procedures used to capture cardiovascular disease and heart disease**

| **Condition or Procedure Code** | **Condition or Procedure Name** |
| --- | --- |
| **ISCHEMIC HEART DISEASE** | |
| 410 | Acute myocardial infarction |
| 411 | Other acute and subacute forms of ischemic heart disease |
| 412 | Old myocardial infarction |
| 413 | Angina pectoris |
| 414 | Other forms of chronic ischemic heart disease |
| 429 | Cardiovascular disease, heart disease unspecified |
| **CEREBROVASCULAR DISEASE** | |
| 430 | Subarachnoid hemorrhage |
| 431 | Intracerebral hemorrhage |
| 433 | Occlusion and stenosis of precerebral arteries |
| 434 | Occlusion of cerebral arteries |
| 435 | Transient cerebral ischemia |
| 436 | Acute, but ill-defined, cerebrovascular disease |
| **PERIPHERAL VASCULAR DISEASE** | |
| 440 | Atherosclerosis |
| 443 | Other peripheral vascular disease |
| **HEART FAILURE** | |
| 425 | Cardiomyopathy |
| 428 | Heart failure |
| **OPERATIONS OF HEART VESSELS OR ENDOVASCULAR PROCEDURES** | |
| 36.0 | Removal Of Coronary Artery Obstruction And Insertion Of Stent(s) |
| 36.1 | Bypass Anastomosis For Heart Revascularization |
| 36.2 | Heart Revascularization By Arterial Implant |
| 36.3 | Other Heart Revascularization |
| 38.1 | Endarterectomy |
| 38.8 | Other Surgical Occlusion Of Vessels |
| 39.29 | Other (Peripheral) Vascular Shunt Or Bypass: |
| 39.50 | Angioplasty Of Other Non-Coronary Vessel |
| 39.7 | Endovascular Repair Of Vessel |
| 39.90 | Insertion Of Non-Drug-Eluting, Non-Coronary Artery Stent |
| V45.8 | Aortocoronary bypass or Percutaneous transluminal coronary angioplasty status |
| 00.4 | Adjunct Vascular System Procedures |
